# Voluntary torque production is unaffected by changes in local thermal sensation during normothermia and hyperthermia

**DOI:** 10.1113/EP090644

**Published:** 2023-02-20

**Authors:** Ralph Joseph Frederick Hills Gordon, Neale Anthony Tillin, Ceri Elen Diss, Christopher James Tyler

**Affiliations:** ^1^ Department of School of Life and Health Sciences University of Roehampton London UK; ^2^ School of Sport Science and Physical Activity University of Bedfordshire Bedford UK; ^3^ Faculty of Science and Engineering, School of Psychology and Sport Science Anglia Ruskin University Cambridge UK

**Keywords:** maximum voluntary contraction, neural drive, perceived thermal strain

## Abstract

This study investigated altered local head and neck thermal sensation on maximal and rapid torque production during voluntary contractions. Nine participants completed four visits in two environmental conditions: at rectal temperatures ∼39.5°C in hot (HOT; ∼50°C, ∼39% relative humidity) and ∼37°C in thermoneutral (NEU; ∼22°C, ∼46% relative humidity) conditions. Local thermal sensation was manipulated by heating in thermoneutral conditions and cooling in hot conditions. Evoked twitches and octets were delivered at rest. Maximum voluntary torque (MVT), normalised surface electromyography (EMG) and voluntary activation (VA) were assessed during brief maximal isometric voluntary contractions of the knee extensors. Rate of torque development (RTD) and EMG were measured during rapid voluntary contractions. MVT (*P* = 0.463) and RTD (*P* = 0.061) were similar between environmental conditions despite reduced VA (−6%; *P* = 0.047) and EMG at MVT (−31%; *P* = 0.019). EMG in the rapid voluntary contractions was also lower in HOT versus NEU during the initial 100 ms (−24%; *P* = 0.035) and 150 ms (−26%; *P* = 0.035). Evoked twitch (+70%; *P* < 0.001) and octet (+27%; *P* < 0.001) RTD during the initial 50 ms were greater in the HOT compared to NEU conditions, in addition to a faster relaxation rate of the muscle (−33%; *P* < 0.001). In conclusion, hyperthermia reduced neural drive without affecting voluntary torque, likely due to the compensatory effects of improved intrinsic contractile function and faster contraction and relaxation rates of the knee extensors. Changes in local thermal perception of the head and neck whilst hyperthermic or normothermic did not affect voluntary torque.

## Abstract (highlights)

- **What is the central question of this study?**
  Hyperthermia reduces the human capacity to produce muscular force, which is associated with decreased neural drive: does mitigating a reduction in neural drive by altering localised thermal sensation help to preserve voluntary force output?
- **What is the main finding and its importance?**
  Altering thermal sensation by cooling and heating the head independent of core temperature did not change neural drive or benefit voluntary force production. Head cooling did slow the rate of rise in core temperature during heating, which may have practical applications in passive settings.

## 1 INTRODUCTION

Maximum voluntary torque (MVT) is used to assess the capacity of the neuromuscular system (Morrison et al., 2004; Périard et al., 2014; Racinais et al., 2008; Ross et al., 2012; Thomas et al., 2006; Todd et al., 2005) but its functional relevance has been questioned due to the time it takes to reach MVT (>125 ms) when contracting from rest (Tillin et al., 2012, 2018). Voluntary rate of torque development (RTD) measures the ability to produce torque rapidly and so is considered more functionally relevant than MVT during activities such as sprinting (Tillin et al., 2013a), joint stabilisation (Domire et al., 2011; Krosshaug et al., 2007) and balance recovery (Behan et al., 2018; Izquierdo et al., 1999). It is widely documented that MVT decreases with increases in core body temperature during brief (≤5 s) (Gordon et al., 2021; Périard et al., 2014; Racinais et al., 2008; Ross et al., 2012; Thomas et al., 2006; Todd et al., 2005) and prolonged (10–120 s) (Morrison et al., 2004; Périard et al., 2014; Racinais et al., 2008; Todd et al., 2005) maximal voluntary contractions (MVCs) with the magnitude of decline in MVT greater during prolonged voluntary efforts compared to brief contractions. However, the effects of high core body temperature on voluntary RTD are less well‐known.

Our laboratory recently showed that voluntary RTD was preserved during high thermal strain (disruption to homoeostasis by stressing the thermoregulatory systems) despite declines in MVT (Gordon et al., 2021). The difference in responses is likely explained by the distinct neural and contractile mechanisms that determine MVT and RTD (Folland et al., 2014). Our recent study (Gordon et al., 2021) found that neural drive (descending voluntary neural input from the central nervous system) decreased with high rectal temperature, at the force plateau (where MVT is measured) and during the rising force–time curve (where RTD is measured). Whilst these declines in neural drive likely caused the reduction in MVT we observed, consistent with previous findings (Morrison et al., 2004; Nybo & Nielsen, 2001; Périard et al., 2014; Racinais et al., 2008; Ross et al., 2012; Thomas et al., 2006; Todd et al., 2005), they could not explain why RTD was preserved, despite neural drive being an important determinant of RTD (Folland et al., 2014). The preservation of voluntary RTD was likely caused by the faster intrinsic contractile properties of the muscle, because of increased muscle temperature (de Ruiter & de Haan, 2000; de Ruiter et al., 1999; Dewhurst et al., 2005), countering the reduction in neural drive. Conceivably, if reductions in neural drive with high core temperature can be mitigated, the benefits of faster contractile properties caused by increases in muscle temperature may record an improvement in voluntary RTD.

One way of potentially attenuating the decline in neural drive in the heat is by decreasing the magnitude of perceived thermal strain through peripheral cooling. Skin temperature can influence human thermal behaviour via local afferent feedback (Schlader et al., 2011a) and changes in skin temperature (e.g., increase due to the ambient environment) can modulate thermal sensation (subjective ratings of the thermal intensity of the surrounding environment) independent of core temperature (Attia & Engel, 1981; Mower, 1976). This is particularly the case when cooling the skin of the head and neck region in hot ambient conditions (Cotter & Taylor, 2005). Reductions in perceived thermal strain, for example, from neck cooling, can improve subsequent exercise performance/capacity in the heat (Sunderland et al., 2015; Tyler & Sunderland, 2011a, 2011b; Tyler et al., 2010). The mechanism for this effect may be associated with an attenuation in hyperthermia‐induced reductions in the neural drive (Gordon et al., 2020; Racinais et al., 2008). An attenuation in the hyperthermia‐induced reduction in neural drive may, therefore, limit declines in MVT and, coupled with faster contractile properties due to a warmer muscle, potentially increase voluntary RTD, during high thermal strain.

If cooling the head when hyperthermic maintains or even improves neuromuscular function by decreasing the perception of thermal strain (reducing thermal sensation and feeling cooler), it is conceivable that the opposite may happen if the perception of thermal strain is increased by heating the head whilst normothermic. There is preliminary evidence of this with non‐thermal warming stimuli (e.g., capsaicin solution) applied to the face while normothermic, decreasing thermal comfort (TC; subjective affective rating of how thermally comfortable the surrounding environment is) and impairing self‐paced exercise (Schlader et al., 2011a, 2011b). Recent data also show that simulated sunlight exposure to the head and neck can impair cognitive function and some motor performance tasks (Piil et al., 2020). To the authors’ knowledge, the use of local thermal warming stimuli, that is, whole head heating, has not been directly investigated on MVT and RTD. Both skin and core body temperatures can influence thermoregulatory behaviour (Flouris & Cheung, 2009; Schlader et al., 2011b). Therefore, directly heating the whole head region should increase thermal sensation (i.e., feeling hotter) and should be a sufficient stimulus to exacerbate the perception of thermal strain while normothermic, which theoretically may reduce neural drive, MVT and voluntary RTD. The comparison of the effects of heating and cooling of the head and neck regions and the subsequent expected alterations to thermal perception may provide further evidence of the contribution of behavioural thermoregulation to the modulation of voluntary force output in hot and temperate conditions. The ability to modulate force output in hot conditions potentially translates beyond exercise performance in the heat, for example, to occupational or military settings, where the ability to perform physical work, specifically, rapid and forceful muscle contractions, is of potential importance.

The aim of this study was to investigate the effect of altered head and neck thermal sensation on MVT, voluntary RTD and their neuromuscular determinants in hyperthermic and normothermic participants. It was hypothesised that improved local perception of thermal sensation via whole‐head cooling during whole‐body hyperthermia would (i) attenuate the expected decline in MVT by preserving neural drive, and (ii) enable participants to benefit from faster contractile properties and so experience increased voluntary RTD, relative to no cooling. Conversely, it was hypothesised that exacerbated perceptions of local thermal sensation via whole head heating while normothermic would decrease both MVT and voluntary RTD, by lowering neural drive compared to no heating.

## 2 METHODS

### 2.1 Ethical approval

All participants were informed of any risks and discomforts associated with the experiment before giving their written informed consent, in accordance with the latest iteration of the *Declaration of Helsinki*, except for registration in a database. Experimental procedures were approved by the Ethical Advisory Committee of the University of Roehampton (LSC 18/242).

### 2.2 Participants

Ten healthy, physically active individuals (*n* = 3 females) volunteered. One male participant voluntarily withdrew from the study because they were unable to tolerate the hot ambient conditions; therefore, data are for *n* = 9. Power analysis was performed for sample size estimation (Gpower 3.1), based on data from Gordon et al. (2021) (*n* = 9) comparing hyperthermia‐induced decreases in EMG_MVT_ at rectal temperature (*T*_re_) 39.5°C compared to ∼37°C, and a large effect (0.14) using η_p_^2^. With an α = 0.05 and β = 0.80, the projected sample size needed was approximately *n* = 10. Participants mean (±SD) age, body mass and stature were 26.6 ± 3.6 years, 71.9 ± 13.4 kg, and 174.6 ± 7.8 cm. Prior to testing, participants confirmed that they had not been exposed to ambient temperatures exceeding 25°C for the 3 weeks prior to participation. To control for the possible impact of variations in hormone levels associated with the menstrual cycle on neuromuscular function (Ansdell et al., 2019) and core body temperature (Baker et al., 2020), female participants began the experimental trials during the early follicular phase (3–5 days after the onset of menstruation) of their self‐reported menstrual cycle and all trials were completed within 2 weeks of starting the first experimental trial. All participants were instructed to refrain from any strenuous physical activity and alcohol consumption for 24 h, and caffeine 12 h prior to each visit to the laboratory.

### 2.3 Overview

Participants visited the laboratory to complete a thorough familiarisation of all the neuromuscular measurements, before returning on four separate occasions (consecutive visits separated by 5 ± 2 days) to complete experimental trials in a walk‐in environmental chamber (Weiss Technik, Loughborough, UK). The experimental trials were conducted at the same time of day for each participant (±13 min), in a randomised order. Two trials were conducted in thermoneutral conditions (∼22°C, ∼46% relative humidity) and two were conducted in hot conditions (∼50°C, ∼39% relative humidity). In each trial, participants completed one set of the same neuromuscular assessment protocol with their preferred leg, using the same protocol as detailed in Gordon et al. (2021). In thermoneutral conditions, this occurred at a pre‐determined time point, 80 min after collecting resting thermoregulatory, cardiovascular and perceptual measurements (see ‘Thermoregulatory, cardiovascular and perceptual responses’); and in the hot conditions at a *T*_re_ of ∼39.5°C.

### 2.4 Protocol

**FIGURE 1:**
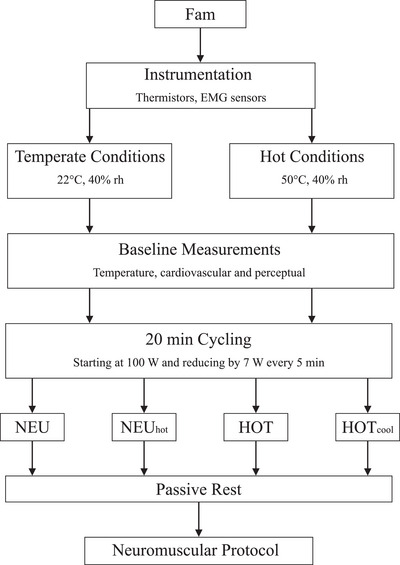
Overview of the experimental study protocol. See sections ‘NEU and NEU_hot_’ and ‘HOT and HOT_cool_’ for specific protocol details. Fam (familiarisation) occurred ∼5 days before participants returned to the laboratory to complete the first experimental trial. rh, relative humidity.

At the start of each experimental trial, participants were instrumented with thermistors and EMG electrodes, before entering the walk‐in environmental chamber. Participants sat quietly on a cycle ergometer (Monark 847E, Vansbro, Sweden) for 2 min before resting thermoregulatory, cardiovascular and perceptual responses were recorded. Participants then performed 20 min of cycling exercise (starting at 100 W and then reducing by 7 W every 5 min) to facilitate internal heat storage, without inducing fatigue from the exercise before the specific experimental trial protocols were followed (NEU and NEU_hot_ and HOT and HOT_cool_). At the end of each experimental trial, the neuromuscular assessment protocol was completed before participants exited the environmental chamber and cooled in the temperate ambient conditions of the laboratory (∼21°C). Once *T*_re_ had returned to 38°C participants recorded a dry, nude body mass. See Figure 1 for a protocol overview.

#### 2.4.1 NEU and NEU_hot_

**FIGURE 2:**
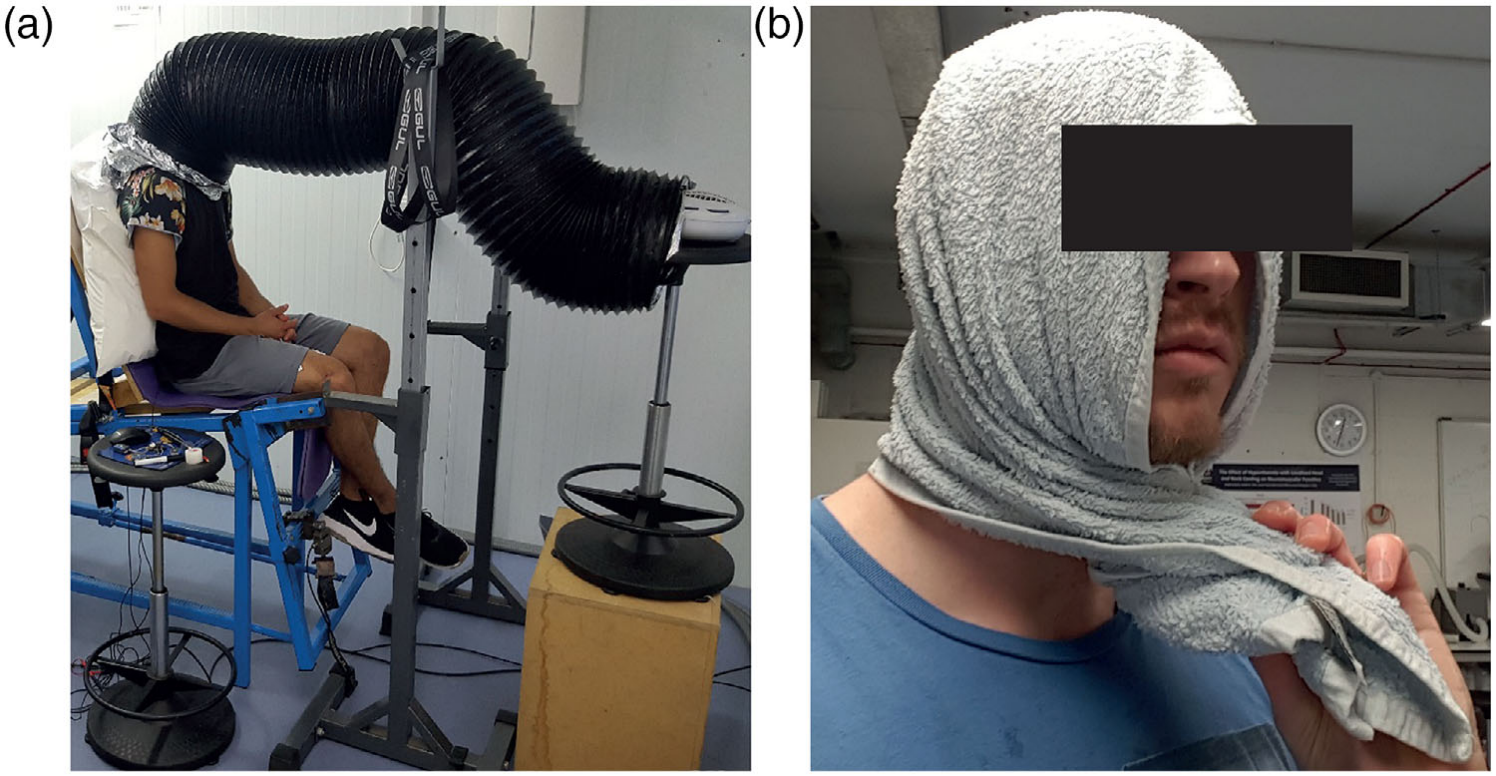
Experimental set up (a) for NEU and NEU_hot_ showing the ventilator duct and fan heating unit, and (b) an example towel used during HOT_cool_.

Two trials were conducted in thermoneutral ambient conditions: thermoneutral control (NEU) and thermoneutral with head and neck heating (NEU_hot_). Following the cycling at the start of the trial, participants moved to the isometric strength testing chair where they were seated but not strapped in. To isolate head and neck heating in NEU_hot_ a flexible ventilator duct, measuring 31.5 cm × 3 m (Fans4Less Ltd, SwiftAir combi flexible duct, Earlswood, UK) was placed over the whole head (Figure 2a). The flexible duct was suspended via strapping attached to two squat rack stands placed on either side of the isometric chair. Directly in front of participants was an electric fan heater (Model: FH950E, Honeywell International Inc., Morris Plains, NJ, USA) blowing hot air (∼1.4 m s^−1^). The flexible duct was suspended, so a slight bend was created midway along the tubing to ensure that air was not blowing directly into the participant's face. Participants wore safety glasses to protect the eye region from heat irritation. The ambient conditions inside the flexible duct were measured by reversing and securing an additional thermistor to the forehead. An emergency foil blanket was folded and wrapped around the neck of the participant to minimise heat loss from the ventilator duct and heat exposure to the upper body during NEU_hot_, and during NEU to replicate conditions of NEU_hot_. During NEU, participants were seated in the same set‐up as in NEU_hot_; however, the electric fan heater was not turned on. Ambient temperatures inside the duct during the neuromuscular assessment protocol were 33.6 ± 0.7°C (NEU) and 47.8 ± 4.3°C (NEU_hot_). Participants remained seated in the isometric strength chair for 60 min before performing the neuromuscular assessment protocol. To minimise discomfort from sitting in the rigid strength testing chair, foam matting and pillows were provided for participants to sit on. These were removed prior to the neuromuscular assessment protocol. The 60 min time was chosen to match an estimated time to reach the target *T*_re_ in the hot ambient conditions (HOT and HOT_cool_), based on a mean Δ*T*_re_ established in pilot testing (0.03°C min^−1^). The ventilator duct remained in place throughout the neuromuscular assessment protocol for both NEU and NEU_hot_.

#### 2.4.2 HOT and HOT_cool_

Two trials were also conducted in hot ambient environmental conditions: hot (HOT) and hot with head and neck cooling (HOT_cool_). In both trials, participants donned an impermeable rain jacket and trouser ensemble (to facilitate internal heat storage) before entering the environmental chamber. Following the cycling, participants remained at rest on an adjustable bed passively heating in either a seated or semi‐recumbent position. Just prior to the target *T*_re_ of ∼39.5°C, participants moved to the isometric strength chair to perform the neuromuscular assessment protocol. To account for the expected rise in *T*_re_ in the hot ambient conditions, the neuromuscular assessment protocol began at a *T*_re_ of 39.4 ± 0.1°C (Table 1), so that mean *T*_re_ during the neuromuscular assessment protocol would be ∼39.5°C. During HOT_cool_, a towel (77 × 46 cm), which had been soaked in water with crushed ice, was wrapped around the head and neck also partially covering the face and cheeks immediately after the cycling exercise finished (Figure 2b). We covered the head, neck and part of the face, to maximise the possibility of alleviating thermal sensation. The towel was changed at regular intervals and worn for the remainder of the trial and during the neuromuscular assessment protocol. The frequency at which the towel was changed was determined by the participant's local thermal sensation (TS_head_), with a rating of ≥3.5 (between feeling ‘cool’ and ‘comfortable’) initiating a replacement towel, or every 2.5 min if the participant's subjective rating was higher. Participants were blinded to the true aim of the study, and therefore not aware that thermal sensation was an important dependent variable. The mean passive heating time to the target *T*_re_ trial was ∼44 min (HOT) and 77 min (HOT_cool_).

**TABLE 1:** Thermoregulatory, cardiovascular and perceptual responses measured at the start and finish of the neuromuscular assessment protocol (then averaged to give a mean value) in four different conditions: thermoneutral control (NEU), NEU with head and neck heating (NEU_hot_), hot (HOT) and hot with head and neck cooling (HOT_cool_).

Parameter	NEU	NEU_hot_	HOT	HOT_cool_
Thermoregulatory				
*T*_re_ (°C)	37.0 ± 0.3	37.1 ± 0.2	39.6 ± 0.1^a^, ^b^	39.4 ± 0.1^a^, ^b^
$\bar{T}$_sk_ (°C)	32.2 ± 0.8	33.4 ± 0.8^c^	39.8 ± 0.5^c^	37.5 ± 0.5^c^
*T*_head_ (°C)	34.9 ± 0.3	43.5 ± 1.9^c^	40.7 ± 0.8^c^	35.2 ± 2.2
$\bar{T}$_neck_ (°C)	33.5 ± 0.7^b^, ^d^	41.2 ± 1.8	40.0 ± 0.4	32.4 ± 2.9^b^, ^d^
Cardiovascular				
HR (beat min^−1^)	74 ± 9	85 ± 14	146 ± 19^a^, ^b^	144 ± 14^a^, ^b^
Perception				
TC	1.1 ± 0.2	1.6 ± 0.6	3.8 ± 0.4^a^, ^b^	2.9 ± 0.9^a^, ^b^
TS_body_	3.8 ± 0.4	4.2 ± 0.9	7.7 ± 0.4^a^, ^b^	6.8 ± 1.0^a^, ^b^
TS_head_	4.1 ± 0.4	6.2 ± 0.8^a^	7.7 ± 0.4^c^	5.0 ± 1.8

Variables are rectal temperature (*T*_re_), mean weighted skin temperature ($\bar{T}$_sk_), head temperature (*T*_head_), mean neck temperature ($\bar{T}$_neck_), heart rate (HR), thermal comfort (TC), thermal sensation of the whole‐body (TS_body_) and thermal sensation of the head and neck (TS_head_). Data are means ± SD for *n* = 8 for *T*_head_, and *n* = 9 for all other variables. Significant (*P* < 0.05) post‐hoc paired differences for condition are denoted by the following:

^a^ different from NEU.

^b^ different from NEU_hot_.

^c^ different between all conditions.

^d^ different from HOT.

#### 2.4.3 Neuromuscular assessment protocol

The same neuromuscular assessment protocol was completed in each experimental trial. Time to complete the set was 343 ± 37 s (mean of all trials). The neuromuscular assessment protocol involved a series of involuntary and voluntary contractions as detailed in the protocol of Gordon et al. (2021) and performed in the order described below.

#### 2.4.4 Twitch and octet

Two twitch and two octet electrically evoked contractions were delivered 20 s apart at rest. The maximal M‐wave (*M*_max_) was calculated as the mean M‐wave response (peak–peak amplitude of the EMG signal) from the two evoked twitches. Both twitch and octet responses were analysed for peak torque (PT), RTD during the initial 50 ms from contraction onset (RTD_0–50_), peak rate of torque development (pRTD), time to peak torque (TPT) and half‐relaxation time (½RT). Dependent variables were averaged across the two twitch or two octet contractions to obtain mean values.

#### 2.4.5 Rapid voluntary contractions

Participants performed 10–15 rapid contractions, in which they were instructed to push as ʻfast and hard' as possible for ∼1.5 s (Tillin et al., 2010), with emphasis on the ʻfast' element of the contraction. For each rapid contraction, participants were encouraged to, as quickly as possible, exceed 80% of the MVT determined in the familiarisation session, with failure to achieve this target resulting in a repeated effort. In addition, any rapid contractions with discernible countermovement or pre‐tension prior to force onset were also repeated. A 5–10 s recovery was allotted between contractions, and participants were instructed to relax their leg as quickly as possible before the next effort. Baseline force was displayed on a sensitive scale on a computer monitor in front of the participants to provide biofeedback on the occurrence of any countermovement or pre‐tension. The slope of the force–time curve (25 ms time constant) was also displayed. The three rapid voluntary contractions with no countermovement or pre‐tension (change in baseline force >0.5 N of the mean, during the 100 ms prior to force onset) and the highest pRTD were used for analysis. Voluntary RTD was measured during three different time epochs: 0–50 ms (RTD_0–50_), 0–100 ms (RTD_0–100_) and 0–150 ms (RTD_0–150_). The root mean squared (RMS) amplitude of the signal at each EMG site was assessed over 0–50 ms (EMG_0–50_), 0–100 ms (EMG_0–100_) and 0–150 ms (EMG_0–150_), normalised to *M*_max_ at the same EMG site, and averaged across the three EMG sites to give a mean value for the quadriceps muscles. Dependent variables were mean averaged across the three rapid contractions selected for analysis. Torque and EMG signal onsets (voluntary and evoked) were identified using visual identification, which is considered the ʻgold standard' of signal onset determination compared to automated detection methods (Tillin et al., 2013b), as per the standardised protocol of Tillin et al. (2010).

#### 2.4.6 Maximal voluntary contractions

Participants performed three MVCs (3–5 s), separated by 30‐s rest, in which they were instructed to push as ʻhard' as possible. At the plateau of the second MVC, two superimposed involuntary doublet contractions were evoked 2 s apart, followed by a doublet contraction evoked at rest 2–5 s after the MVC. MVT was defined as the greatest voluntary (i.e., not due to superimposed doublet stimulation) torque recorded in any of the rapid contractions or MVCs. To assess neural drive at MVT (i.e., at the MVC plateau), the amplitudes of the superimposed doublets were used to determine voluntary activation (VA) using the following formula:

$$\mathrm{VA}\;\left(\%\right)=\left[1-\left(\frac{D_{\sup}}{D_{\mathrm{con}}}\right)\right]\times 100$$

where *D*_sup_ represents the superimposed doublet amplitude and *D*_con_ the potentiated doublet amplitude evoked at rest after the MVC. VA was calculated from one of the superimposed doublets (whichever was delivered at the greatest torque value) during the same MVC. Neural drive was also assessed from the RMS amplitude over a 500‐ms epoch surrounding MVT (250 ms either side, without the influence of artefact from electrical stimulation), normalised to maximal *M*_max_, and averaged across the three EMG sites to give a mean value for the whole quadriceps muscle (EMG_MVT_).

### 2.5 Measurements

#### 2.5.1 Torque

Seated in a custom‐built strength testing chair (Maffiuletti et al., 2016), participants were securely fastened with a waist belt and shoulder straps with hip and knee angles fixed at 100° and 105°, respectively (180° defined full extension). An ankle strap, in series with a strain gauge, load cell (FSB Universal Cell 1.5 kN, Force Logic, Reading, UK) was secured 4 cm proximal to the medial malleolus, with the load cell aligned perpendicular to the tibia during knee extension. The force signal was amplified (×375) and sampled at 2000 Hz via an analog‐to‐digital converter (Mirco3 1401, CED, Cambridge, UK) and PC using Spike2 software (Spike 2 Version 8, CED). A computer monitor in view of the participant provided real‐time biofeedback. In the thermoneutral trials, detailed verbal feedback was provided because participants were unable to see the computer monitor. Offline, the force signal was filtered using a fourth‐order low‐pass Butterworth filter with a cut‐off frequency of 500 Hz, corrected for limb weight and multiplied by the external moment arm (distance between the lateral knee joint space and the centre point of the ankle strap) to calculate knee extension torque.

#### 2.5.2 EMG

Following preparation of the skin (shaving, light abrasion, and cleaning using 70% ethanol), a bipolar silver–silver chloride gel‐electrode configuration (2 cm diameter and 2 cm inter‐electrode distance; Dual Electrode, Noraxon, Scottsdale, AZ, USA) was placed over the belly of the rectus femoris (RF), vastus lateralis (VL) and vastus medialis (VM). Electrode configurations were placed parallel to the presumed orientation of the muscle fibres at specific distances from the greater trochanter to the lateral knee joint space (47 ± 7% (RF), 74 ± 4% (VL) and 83 ± 4% (VM)). The placement of electrodes was conducted by the same investigator throughout all trials and established during familiarisation, with the position of each electrode marked on the skin using a permanent pen. Participants were instructed not to actively wash these marks off between trials so that marks could be re‐applied, and electrodes relocated in the same location at the beginning of the experimental trials. Each EMG signal was amplified (×500; 10–500 Hz bandwidth), transmitted wirelessly to a desktop receiver (TeleMyoDTS, Noraxon), and sampled (2000 Hz) in synchronisation with force via the same analog‐to‐digital converter utilising Spike2 software. In off‐line analysis, the EMG signals were bandpass‐filtered between 6 and 500 Hz using a fourth‐order Butterworth digital filter and aligned with the force signal to correct for the 156 ms delay inherent in the Noraxon TeleMyoDTS system.

#### 2.5.3 Electrical stimulation

Electrical square‐wave pulses (200 μs duration) delivered over the femoral nerve (DS7AH Constant Current Stimulator, Digitimer, Welwyn Garden City, UK) were used to evoke twitch (single pulse), doublet (two pulses at 100 Hz) and octet (eight pulses at 300 Hz) contractions. The anode (Rubber electrode 10 × 7 cm, EMS Physio Ltd, Wantage, UK) was secured by surgical tape (Transpore, 3M, Bracknell, UK) to the skin over the greater trochanter. The cathode stimulation probe (1 cm diameter lint tip; S1 Compex Motor PointPen, Digitimer), which protruded 2 cm from the centre of a custom‐built plastic base (4 × 3 cm), was placed over the femoral nerve in the femoral triangle. The greatest evoked peak twitch force in response to a submaximal current determined the precise placement of the cathode, where it was taped in place. The electrical current was then increased incrementally by 20 mA until there was a plateau in both twitch peak force and peak‐to‐peak M‐wave amplitude at each EMG site. This current was increased by a further 20% (supramaximal) to ensure that all stimulations were eliciting a maximal involuntary response, and this current (110 ± 29 mA) was used for all twitch, doublet and octet contractions thereafter. The cathode position and supramaximal stimulation intensity were determined for each participant in the familiarisation session and then kept constant for the experimental trials, with the cathode position being marked on the skin with permanent ink and maintained by participants to ensure accurate relocation between trials.

#### 2.5.4 Thermoregulatory, cardiovascular and perceptual responses

A rectal thermistor (REC‐U‐VL30, Grant Instruments, Cambridge, UK) was self‐inserted ∼10 cm past the anal sphincter to measure *T*_re_. Seven wireless skin thermistors (iButton DS1922L; Maxim/Dallas Semiconductor Corp., Texas, USA) were applied to the skin with a transparent dressing (Tegaderm, 3M, St Paul, MN, USA) and secured with surgical tape for the assessment of local skin temperature. Mean weighted skin temperature ($\bar{T}$_sk_) was calculated (Ramanathan, 1964) from four skin sites located on the right side of the body: suprasternal notch, flexi carpi radials, gastrocnemius and RF. Mean neck temperature ($\bar{T}$_neck_) was measured from two skin thermistors placed on either side of the spinal midline at the third/fourth cervical vertebrae. Temperature of the head (*T*_head_) was measured from one skin thermistor placed on the forehead. Due to a technical error, the *T*_head_ data are for *n* = 8. Whole body thermal sensation (TS_body_) and local thermal sensation of the head and neck (TS_head_) were rated using a nine‐point scale from 0 (unbearably cold) to 8 (unbearably hot) with 4 as comfortable (neutral) (Young et al., 1987). Whole body TC was measured using a four‐point scale from 1 (comfortable) to 4 (very uncomfortable) (Gagge et al., 1967). All thermoregulatory and perceptual measurements were recorded at 2.5‐min intervals up to when the neuromuscular assessment protocol commenced. Thereafter, responses were recorded at the start and end of the protocol only.

#### 2.5.5 Fluid loss

Participants consumed 500 ml of water 2 h before each experimental trial. Pre‐trial hydration status was assessed from a mid‐stream urine sample. Euhydration was assumed for all (urine specific gravity ≤1.020). Nude body mass was recorded pre‐ and post‐, and water (non‐chilled) was provided ad libitum during each experimental trial. After correcting for fluid intake and urine output, body mass changes were used to estimate sweat loss.

### 2.6 Statistical analyses

All data were assessed for normality of distribution and violations of sphericity were corrected for using the Greenhouse–Geisser adjustment when appropriate. Descriptive data are reported as means ± SD. A two‐way repeated measures ANOVA was used to assess the influence of condition and time (4 conditions × 9 time points) on *T*_re_, $\bar{T}$_sk_, heart rate (HR), *T*_head_, $\bar{T}$_neck_, TC, TS_body_ and TS_head_. Because the trial time lengths differed in HOT and HOT_cool_, but all trials were on a continuous scale, that is, passive heating followed immediately after the 20‐min cycling exercise, passive heating is expressed as a percentage of trial time. For all other dependent variables, a one‐way repeated measures ANOVA was used to assess the effect of condition (NEU vs. NEU_hot_ vs. HOT vs. HOT_cool_). Following a significant *F*‐value, pairwise differences were identified using stepwise Bonferroni‐corrected paired Student's *t*‐test. Effect sizes for paired comparisons were calculated using Cohen's *d* and interpreted as small (0.2), medium (0.5) or large (0.8) (Cohen, 1988). The α‐level was set at *P* < 0.05. Statistical analysis was completed using SPSS Statistics version 26 (IBM Corp., Armonk, NY, USA).

## 3 RESULTS

### 3.1 Temperature and HR

**FIGURE 3:**
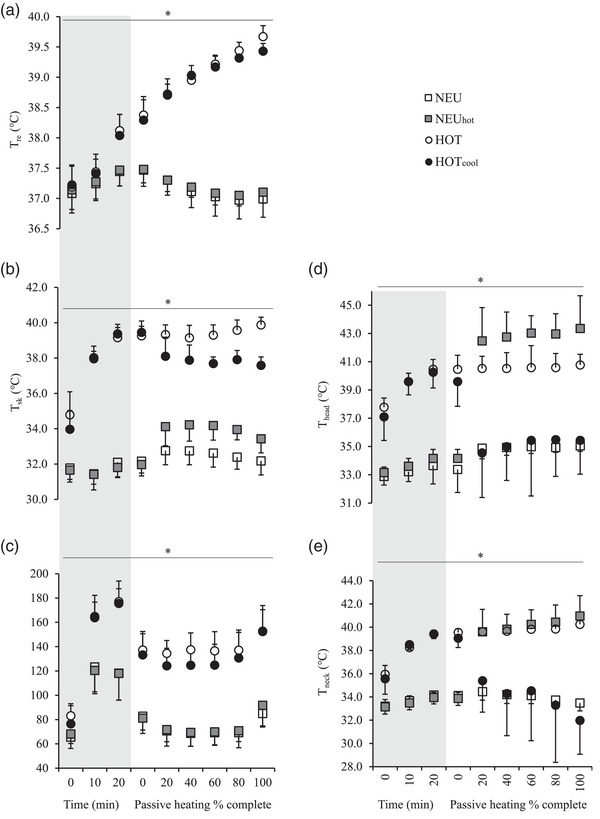
Responses from (a) rectal temperature (*T*_re_), (b) mean weighted skin temperature ($\bar{T}$_sk_), (c) heart rate (HR), (d) head temperature (*T*_head_) and mean neck temperature ($\bar{T}$_neck_) in four different conditions, thermoneutral control (NEU), NEU with head and neck heating (NEU_hot_), hot (HOT) and hot with head and neck cooling (HOT_cool_). All trials were completed on a continuous scale. The grey area denotes responses during the 20‐min cycling, but because trial lengths differed during HOT and HOT_cool_, data are reported as a percentage of trial time during passive heating. Time to target *T*_re_ was ∼44 min (HOT) and 77 min (HOT_cool_). Data are means ± SD, *n* = 8 for *T*_head_ and *n* = 9 for all other variables. *Main effect of time (*P* < 0.05).

There were main effects of condition (*P* < 0.001), time (*P* < 0.001) and interaction (*P* < 0.001) on *T*_re_, $\bar{T}$_sk_, *T*_head_, $\bar{T}$_neck_ and HR during the trials, as per the study design. The changes in these responses during each condition are detailed in Figure 3. For brevity, the results section will focus on the between‐condition responses when the neuromuscular assessment occurred. Table 1 illustrates absolute average mean values taken from the start and end of the neuromuscular protocol. There was a main effect of condition on all temperature and cardiovascular variables (*P* < 0.001). *T*_re_ and HR were greater in the two HOT conditions than the two NEU conditions (*P* < 0.001; *d* = 3.7–15.5). $\bar{T}$_sk_ was different between all trial comparisons (*P* ≤ 0.034; *d* = 1.5–11.3), being highest to lowest in the following order HOT > HOT_cool_ > NEU_hot_ > NEU. The cooling and heating protocols effectively changed the local head and neck temperatures, without affecting *T*_re_. Specifically, *T*_head_ and $\bar{T}$_neck_ in NEU_hot_ and HOT were hotter than NEU and HOT_cool_ (Table 1). *T*_head_ was statistically different between all trials (*P* ≤ 0.026; *d* = 1.9–9.3), while $\bar{T}$_neck_ was hotter in NEU_hot_ and HOT than NEU and HOT_cool_ (*P* < 0.001; *d* = 0.9–11.8).

When Δ*T*_re_ was expressed in absolute values, there was a main effect of condition (*P* < 0.001). The mean Δ*T*_re_ was similar between NEU and NEU_hot_ (*P* > 0.999), but slower during HOT_cool_ (0.03 ± 0.01°C min^−1^) than HOT (0.05 ± 0.01°C min^−1^; *P* = 0.002; *d* = 1.4), increasing the mean (+34 min) time to achieve the target *T*_re_ in HOT_cool_.

### 3.2 Perceptual responses

**FIGURE 4:**
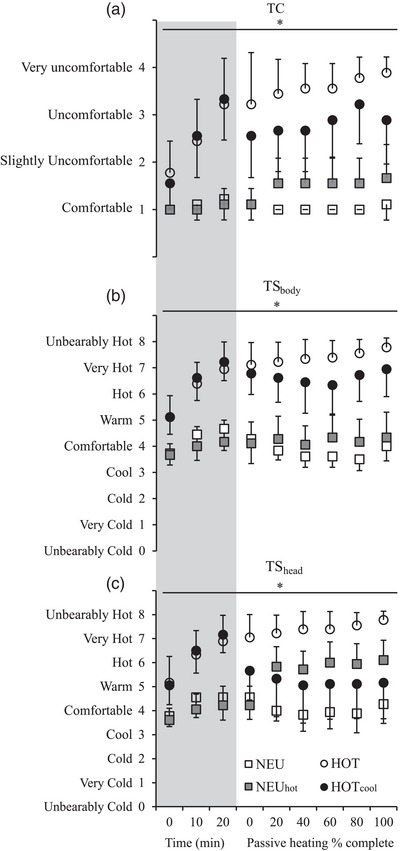
Perceptual responses from (a) thermal comfort (TC), (b) whole‐body thermal sensation (TS_body_), and (c) thermal sensation of the head and neck (TS_head_) in four different conditions; thermoneutral control (NEU), NEU with head and neck heating (NEU_hot_), hot (HOT) and HOT with head and neck cooling (HOT_cool_). All trials were completed on a continuous scale. The grey area denotes responses during the 20‐min cycling, but because trial lengths differed during HOT and HOT_cool_ data are reported as a percentage of trial time during passive heating. Time to target *T*_re_ was ∼44 min (HOT) and 77 min (HOT_cool_). Data are means ± SD for *n* = 9. *Main effect of time (*P* < 0.05).

There were main effects of condition (*P* < 0.001), time (*P* < 0.001) and interaction (*P* < 0.001) on TC, TS_body_ and TS_head_. The changes in these responses during each condition are detailed in Figure 4. Table 1 shows the absolute average mean values taken from the start and end of the neuromuscular protocol. There was a main effect of condition (*P* < 0.001) on all perceptual variables (Table 1). Participants felt more uncomfortable (TC; *P* ≤ 0.016; *d* = 1.7–10.1) and hotter (TS_body_; *P* < 0.001; *d* = 2.7–10.2) in the hot (HOT and HOT_cool_) compared to the temperate conditions (NEU and NEU_hot_). In contrast, TS_head_ was cooler in NEU and HOT_cool_ compared to HOT and NEU_hot_ (*P* ≤ 0.010; *d* = 0.8–8.9).

### 3.3 Estimated sweat rate and body mass change

There was a main effect of condition on estimated sweat rate (*P* < 0.001) and body mass change percentage (*P* < 0.001). Sweat rate was not statistically different between HOT (1.7 ± 0.2 L h^−1^) and HOT_cool_ (1.3 ± 0.4 L h^−1^; *P* = 0.242; *d* = 0.7), but both were greater than NEU (0.2 ± 0.1 L h^−1^) and NEU_hot_ (0.3 ± 0.1 L h^−1^), with NEU_hot_ also exhibiting a greater sweat response than NEU (*P* ≤ 0.028; *d* = 1.0–3.7). There was a greater change in body mass percentage in HOT (0.5 ± 0.4%) compared to NEU (0.2 ± 0.3%; *P* = 0.042; *d* = 1.1), but it was not statistically different in all other comparisons (NEU_hot_: 0.3 ± 0.3%; HOT_cool_: 1.0 ± 0.7%; *P* ≥ 0.181; *d* = 0.3–1.5).

### 3.4 Voluntary torque

**FIGURE 5:**
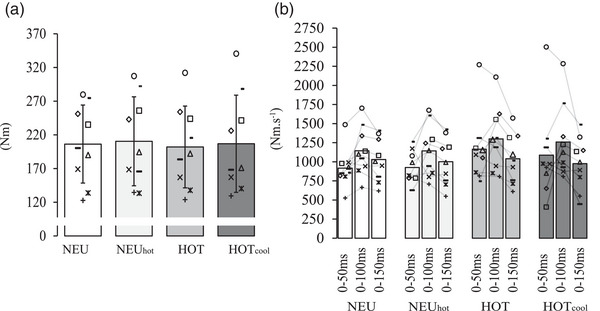
Maximum voluntary torque (MVT; a), and voluntary rate of torque development (RTD; b) in four different conditions, thermoneutral control (NEU), NEU with head and neck heating (NEU_hot_), hot (HOT) and HOT with head and neck cooling (HOT_cool_). Individual data points are presented and means ± SD are for *n* = 9. To improve clarity, SD bars have been omitted for RTD.

There was no effect of condition on MVT (*P* = 0.463, Figure 5a), RTD_0–50_ (*P* = 0.232), RTD_0–100_ (*P* = 0.061) or RTD_0–150_ (*P* = 0.643, Figure 5b).

### 3.5 Neural drive

There was a main effect of condition for EMG_MVT_ (*P* = 0.019) and VA (*P* = 0.047; Table 2), but post‐hoc analysis did not reveal any significant comparisons between conditions for EMG_MVT_ (*P* ≥ 0.145; *d* = 0.2–1.0) or VA (*P* ≥ 0.377; *d* = 0.0–0.5). During the rapid voluntary contractions, there was no main effect of condition on EMG_0–50_ (*P* = 0.064; Table 2), but there was a main effect on EMG_0–100_ (*P* = 0.003) and EMG_0–150_ (*P* = 0.002). Post‐hoc analysis showed that both EMG_0–100_ (*P* = 0.035; *d* = 1.8) and EMG_0–150_ (*P* = 0.035; *d* = 1.8) decreased in HOT compared to NEU. There were no other significant comparisons between conditions (*P* ≥ 0.094; *d* = 0.5–1.5).

**TABLE 2:** Neural drive of the knee extensors during four different conditions: thermoneutral control (NEU), NEU with head and neck heating (NEU_hot_), hot (HOT) and HOT with head and neck cooling (HOT_cool_).

Parameter	NEU	NEU_hot_	HOT	HOT_cool_
MVCs				
EMG_MVT_ (%)	7.8 ± 2.7	7.0 ± 1.9	5.2 ± 2.3	6.5 ± 2.4
VA (%)	88 ± 10	87 ± 14	83 ± 12	82 ± 12
Rapid contractions				
EMG_0–50_ (%)	7.6 ± 1.3	6.8 ± 1.4	5.8 ± 1.0	6.4 ± 1.9
EMG_0–100_ (%)	8.0 ± 1.2	7.6 ± 1.1	6.1 ± 1.0^a^	6.8 ± 1.3
EMG_0–150_ (%)	7.8 ± 1.3	7.3 ± 1.0	5.8 ± 1.0^a^	6.8 ± 1.3

Dependent variables are EMG RMS at maximal voluntary torque (EMG_MVT_) and normalised to *M*_max_, voluntary activation (VA) during maximal voluntary contractions and EMG RMS during the rapid voluntary contractions at three different time epochs, 0–50 ms (EMG_0–50_), 0–100 ms (EMG_0–100_) and 0–150 ms (EMG_0–150_), also normalised to *M*_max_. Data are means ± SD for *n* = 9.

^a^ Different from NEU, significant (*P* < 0.05) post‐hoc paired difference.

### 3.6 Intrinsic contractile properties

There was a main effect of condition (*P* < 0.05) on all twitch parameters (Table 3). Twitch PT was greater in HOT and HOT_cool_ than NEU (*P* ≤ 0.015; *d* = 0.6–0.9), but not statistically different between other trial comparisons (*P* ≥ 0.072; *d* = 0.3–0.6). Twitch RTD_0–50_ was greater in HOT than all other conditions (*P* ≤ 0.036; *d* = 0.4–1.2), while HOT_cool_ was greater than NEU (*P* = 0.001; *d* = 0.8). All other trial comparisons were not statistically different (*P* ≥ 0.078; *d* = 0.3–0.6). Twitch pRTD was greater in HOT and HOT_cool_ than NEU and NEU_hot_ (*P* ≤ 0.025; *d* = 0.9–1.5), but not statistically different between other trial comparisons (*P* ≥ 0.461; *d* = 0.4). Twitch TPT was only significantly faster in HOT versus NEU_hot_ (*P* = 0.031; *d* = 1.3), while all other trial comparisons were not statistically different (*P* ≥ 0.109; *d* = 0.1–1.1). Twitch ½RT was faster in HOT and HOT_cool_ than NEU and NEU_hot_ (*P* ≤ 0.025; *d* = 0.9–1.5), but not statistically different between other trial comparisons (*P* ≥ 0.461; *d* = 0.4).

**TABLE 3:** Responses from evoked supramaximal twitch and octet contractions in the knee extensors at rest, during four different conditions: thermoneutral control (NEU), NEU with head and neck heating (NEU_hot_), hot (HOT) and hot with head and neck cooling (HOT_cool_).

Parameter	NEU	NEU_hot_	HOT	HOT_cool_
Twitch				
PT (N m)	32 ± 13	36 ± 14	47 ± 19^a^	42 ± 18^a^
RTD_0–50_ (N m s^−1^)	488 ± 218	553 ± 228	827 ± 336^b^	710 ± 299^a^
pRTD (N m s^−1^)	1046 ± 337	1181 ± 373	1796 ± 647^a^, ^b^	1591 ± 518^a^, ^b^
TPT (ms)	80 ± 7	82 ± 8	74 ± 6^c^	73 ± 10
½RT (ms)	76 ± 14	77 ± 13	51 ± 11^a^, ^b^	57 ± 10^a^, ^b^
Octet				
PT (N m)	150 ± 51	151 ± 52	163 ± 55	149 ± 43
RTD_0–50_ (N m s^−1^)	1648 ± 528	1677 ± 557	2089 ± 631^a^, ^b^	1912 ± 571^a^
pRTD (N m s^−1^)	3250 ± 974	3295 ± 930	4410 ± 1500^a^, ^b^	3987 ± 1281
TPT (ms)	133 ± 6	135 ± 6	116 ± 8^a^, ^b^	119 ± 21^a^, ^b^
½RT (ms)	76 ± 16	73 ± 10	51 ± 15^a^, ^b^	52 ± 13^a^, ^b^

Dependent variables are peak torque (PT), rate of torque development in the first 50 ms (RTD_0–50_), peak rate of torque development (pRTD), time to peak torque (TPT) and half‐relaxation time (½RT). Data are means ± SD for *n* = 9. Significant (*P* < 0.05) post‐hoc paired differences are denoted by the following:

^a^ different from NEU.

^b^ different between all conditions.

^c^ different from NEU_hot_.

Except for PT (*P* = 0.160), there was a main effect of condition (*P* < 0.001) on all other octet parameters (Table 3). Octet RTD_0–50_ was greater in HOT than NEU and NEU_hot_ (*P* ≤ 0.001; *d* = 0.7–0.8), while HOT_cool_ was greater than NEU (*P* = 0.014; *d* = 0.5). All other trial comparisons were not statistically different (*P* ≥ 0.112; *d* = 0.1–0.4). Octet pRTD was greater in HOT than NEU and NEU_hot_ (*P* ≤ 0.033; *d* = 0.9), but not statistically different between other trial comparisons (*P* ≥ 0.056; *d* = 0.1–0.6). Both octet TPT (*P* ≤ 0.012; *d* = 1.9–2.7) and ½RT (*P* ≤ 0.06; *d* = 1.5–1.8) were faster in HOT and HOT_cool_ than NEU and NEU_hot_. All other trial comparisons were not statistically different (*P* ≥ 0.99; *d* = 0.0–0.4).

## 4 DISCUSSION

The present study aimed to investigate the manipulation of local head and neck thermal sensation independent of core body temperature on the neural and contractile mechanisms responsible for rapid and maximal voluntary torque capacity. Neither hyperthermia nor the manipulation of thermal sensation affected MVT or voluntary RTD, but there was evidence that neural drive was affected by hyperthermia, specifically, decreasing in the heat. During the rapid voluntary contractions, EMG_0–100_ and EMG_0–150_ were shown to decrease in HOT compared to NEU. Nevertheless, the consistent MVT and RTD across conditions, despite effects on neural drive, may partly be explained by a trade‐off in improved intrinsic contractile function (greater twitch/octet torques and faster contraction and relaxation times).

MVT (Figure 5a) and voluntary RTD (Figure 5b) were similar between HOT and NEU. Hyperthermia can be induced by active (e.g., exercise) or passive methods, with the former potentially confounding interpretation of the influence of thermal strain on hyperthermia‐induced reductions in voluntary force output. Studies using active hyperthermia but assessing neuromuscular function on non‐exercised limbs have not shown decreases in maximal voluntary force (Martin et al., 2005; Nybo & Nielsen, 2001; Rattey et al., 2006; Saboisky et al., 2003), whilst studies using passive protocols (e.g., liquid conditioning garments) have demonstrated temperature‐induced declines (Gordon et al., 2021; Morrison et al., 2004; Périard et al., 2014; Racinais et al., 2008; Ross et al., 2012; Thomas et al., 2006; Todd et al., 2005). MVT in the present study appears to corroborate the aforementioned active hyperthermia studies, and contrast with the passive protocols. Whilst the present study employed a low‐intensity cycling exercise bout (mean of all trials, 85.9 ± 0.6 W), it is unlikely that this would have elicited exercise‐induced fatigue to the lower limbs, given the amount of time participants passively rested between finishing the exercise and starting the neuromuscular assessment protocol. One explanation for no change in MVT, and a limitation of the study, is the omission of baseline measurement of neuromuscular function, specifically in the hot ambient conditions. The comparison of MVT between different ambient conditions on different trial days potentially masks any observable decline in MVT. Gordon et al. (2021) recently demonstrated this by showing a 12% (*P* < 0.05) decrease in MVT at *T*_re_ 39.5°C relative to a baseline measure taken at *T*_re_ ∼37°C, in the same trial. However, when MVT at *T*_re_ 39.5°C in the hot ambient conditions was compared to the normothermic control trial, there was no difference (*P* > 0.05) in MVT. We, therefore speculate had there been a baseline measure of MVT in the present study, there would have been an observable hyperthermia‐induced decline in MVT. The similarity in voluntary RTD between HOT and NEU was expected, although there appears to be a subtle increase in RTD during the initial 50 ms from contraction onset when hyperthermic. These data are consistent with recent evidence from our group demonstrating that voluntary RTD does not decrease during high thermal strain (Gordon et al., 2021). The manipulation of local thermal sensation did not affect either MVT or voluntary RTD, which contrasted with our original hypothesis. Whilst torque output was not modified by local changes in thermal sensation, there may have been some differences in both neural drive and the intrinsic contractile properties, which will be discussed below.

MVT and voluntary RTD were unaffected by high thermal strain or the modulation of perception of thermal strain, but neural drive decreased following hyperthermia. There were moderate‐to‐large effect sizes for declines in VA (−6%; *d* = 0.5) and EMG_MVT_ (−31%; *d* = 0.8) during HOT compared to NEU. A hyperthermia‐induced reduction in neural drive at MVT is well‐documented (Morrison et al., 2004; Périard et al., 2014; Racinais et al., 2008; Ross et al., 2012; Thomas et al., 2006; Todd et al., 2005), with recent data also showing a decline in the neural drive during rapid voluntary contractions with high thermal strain (Gordon et al., 2021). The present study supports this finding, with reductions in both EMG_0–100_ (−23%) and EMG_0–150_ (−24%; Table 2) during whole‐body hyperthermia. The authors speculate that the manipulation of TS_head_ from cooling may have had a small (non‐significant) effect (HOT vs. HOT_cool_; EMG_MVT_, *d* = 0.5, EMG_0–50_, *d* = 0.4, EMG_0–100_, *d* = 0.7, and EMG_0–150_, *d* = 0.9) on neural drive compared to no cooling in the heat. The implication is that whole‐head and neck cooling during hyperthermia may attenuate a decrease in the neural drive.

The changes in TS_head_ and the possible effect on the neural drive could be explained in part by the high alliesthesial thermosensitivity of the head and neck regions (Cotter & Taylor, 2005), which has a small surface area but a large effect on thermal sensation and discomfort (Brown & Williams, 1982). A reduction in the neural drive from head and neck heating is plausible, with research conducted on non‐thermal facial heating (Schlader et al., 2011a) increasing perceptual sensations of the heat and decreasing TC, which can reduce cycling capacity (Schlader et al., 2011a). However, the present data do not support this in a predominantly passive heating context.

It is interesting to note that neither TC nor TS_body_ was statistically different within the two environmental conditions (NEU vs. NEU_hot_ and HOT vs. HOT_cool_). This finding is in spite of statistical differences in $\bar{T}$_sk_ for all trial comparisons (Table 1). Cutaneous thermoreceptors are thought to influence thermal sensation (Mower, 1976), and thus if $\bar{T}$_sk_ was different within the environmental conditions, it might be expected that these changes should also be reflected in TS_body_. This may be due to a lack of sensitivity in the psychophysical scales used to assess thermal sensation (Young et al., 1987) and TC (Gagge et al., 1967), where the scale can become quickly ‘saturated’ and no longer provides quantifiable measures of sensations of warmth and how pleasant these may or may not be (Cabanac, 1975; Hensel, 1981). Alternatively, the discrepancy could be linked to anchoring biases (Raccuglia et al., 2018). It may be that the manipulation of TS_head_ in the present study was not sufficient to effect meaningful changes in TC during either NEU_hot_ or HOT_cool_, in part because TC is influenced by core body temperature (Cabanac, 1971). *T*_re_ was 37.1 ± 0.2°C in NEU_hot_ and 39.4 ± 0.1°C in HOT_cool_, which were similar to NEU and HOT, respectively (Table 1), which adds to the growing body of literature that suggests that core body temperature is a key determinant of hyperthermia‐induced declines in the neural drive (Gordon et al., 2021; Morrison et al., 2004; Nybo & Nielsen, 2001; Périard et al., 2014; Racinais et al., 2008; Ross et al., 2012; Thomas et al., 2006; Todd et al., 2005).

Muscle temperature was not recorded in the present study, although it is likely that high thermal strain did cause a rise in muscle temperature and subsequent improvements in the intrinsic contractile properties (de Ruiter & de Haan, 2000; de Ruiter et al., 1999; Dewhurst et al., 2005). These improvements are evidenced by the faster TPT and ½RT in both twitch and octet responses in HOT and HOT_cool_ compared to NEU and NEU_hot_ (Table 3), resulting from improved excitation–contraction coupling and faster cross‐bridge cycling mechanics via an increased rate of myosin–actin attachment (Davies et al., 1982). However, there were subtle differences between HOT and HOT_cool_. Twitch RTD_0–50_ was 14% (*d* = 0.4) lower in HOT_cool_ than HOT, which could partly explain why no increase in voluntary RTD was observed, relative to HOT and in contrast to our original hypothesis, given that twitch RTD_0–50_ is a determinant of early phase rapid torque production (Andersen & Aagaard, 2006; Folland et al., 2014). In addition, octet RTD_0–50_, which is a determinant of middle phase (50–100 ms) rapid torque production (Folland et al., 2014), was greater in HOT than both NEU and NEU_hot_, but in HOT_cool_, statistical significance was only observed compared to NEU_hot_ (Table 3). Last, octet pRTD in HOT_cool_ was not statistically different from either NEU or NEU_hot_ and 7% (*d* = 0.3) lower than HOT. Taken together, these data suggest that the intrinsic contractile properties produced lower involuntary RTD when cooling was applied to the head and neck compared to no cooling.

We have previously observed that high thermal strain creates a compensatory mechanism to preserve voluntary RTD through improved intrinsic contractile function as well as faster contraction and relaxation rates of the muscle. This is achieved via increased muscle temperature, which consequently counteracts the hyperthermia‐induced decrease in the neural drive (Gordon et al., 2021). When we examined skin temperature taken from the RF, it was statistically different between all trial conditions (*P* ≤ 0.020; *d* = 0.9–16.9). Using thigh skin temperature (HOT_cool_; 39.5 ± 1.1°C vs. HOT; 41.0 ± 0.3°C) as a surrogate estimation of muscle temperature, it may be that intrinsic contractile function improved in HOT compared with HOT_cool_ because the head and neck cooling unexpectedly lowered thigh muscle temperature in HOT_cool_.

Head and neck cooling increased the time to achieve the target *T*_re_ (39.5°C) by slowing the Δ*T*_re_ during the passive heating, which resulted in a lower mean session *T*_re_ for HOT (38.4 ± 0.1°C) compared to HOT_cool_ (38.6 ± 0.2°C; *P* = 0.02; *d* = 0.9). The increased heat exposure was reflected in a greater estimated sweat rate in HOT_cool_ compared to HOT; however, ad libitum fluid replacement was adequate to prevent significant changes in body mass percentage, suggesting that hydration status was similar between these conditions. Therefore, head and neck cooling successfully alleviated local perceptions of thermal strain, but at the expense of prolonged heat exposure due to an apparent blunted Δ*T*_re_ in HOT_cool_. The authors speculate this blunted Δ*T*_re_ may be linked to a reduction in brain temperature, although, there is conflicting evidence to suggest that this may not be the case (see review by Marino, 2011). A recent study demonstrated a decrease in the brain (−0.9°C) and rectal (−0.3°C) temperature in healthy individuals (Diprose et al., 2022), suggesting the possibility that cooling the whole head may create a thermal gradient between the brain and body. The authors, therefore, cannot disregard the effects that a reduced brain temperature may have had, although these effects are currently unknown. Diprose et al. (2022) used a powerful cooling system to reduce the temperature of the head; therefore, any decrease in brain temperature in the current study is likely to have been smaller in magnitude in comparison. The increase in the duration of the protocol from cooling presents a further added confounding effect, and future research could extend these findings by focussing on dynamic reductions in skin temperature (de Dear et al., 1993). Nevertheless, data in this study suggest that local cooling or heating of the head–neck region did not inhibit or benefit neural drive, and by extension voluntary torque. This suggests the difference in *T*_re_ between the temperate and hot ambient conditions, rather than the modifications in thermal perception, is a key factor in inhibiting voluntary muscle.

There are some practical implications of the presented findings. Locally cooling the head and neck regions with a sufficient cooling stimulus may not only improve local thermal sensation in the heat, but also slow the rate of rise in core temperature, in a passive setting. This manipulation of local thermal sensation does not affect measures of voluntary torque production in temperate or hot ambient conditions; however, it can slow the rise in core temperature, which may be practically beneficial in scenarios requiring physical exertion for sporting performance, occupational tasks or habitual exposure to the heat. Where interventions are used to reduce sensation and thus declines in neural drive, care should be taken for health reasons, as cooling may disrupt a potential natural protective mechanism to limit metabolic heat production from muscular activity.

### 4.1 Conclusion

In conclusion, increased core temperature reduced neural drive during maximal and rapid voluntary contractions. MVT and RTD were not affected by the changes in neural drive, likely due to a trade‐off with the effects of the heat on the contractile properties. Local thermal sensation in the heat was improved with cooling of the head and neck while heating the same area during normothermia had the opposite effect. The local manipulations used to alter thermal perception had minimal impact on thermal discomfort, accounting for why the neural drive was neither increased with head cooling whilst hyperthermic nor decreased with head heating whilst normothermic.

## AUTHOR CONTRIBUTIONS

The present investigation was conducted at the Sports and Exercise Science Research Centre physiology laboratory, located on the Whitelands campus at the University of Roehampton. Ralph J. F. H. Gordon, Neale A. Tillin, Ceri Diss and Christopher J. Tyler contributed to the conception and design of the study. Ralph J. F. H. Gordon, Neale A. Tillin, Ceri Diss and Christopher J. Tyler contributed to the acquisition, analysis and interpretation of the data. Ralph J. F. H. Gordon drafted the manuscript and Neale A. Tillin, Ceri Diss and Christopher J. Tyler made critical revisions. All authors have read and approved the final version of this manuscript and agree to be accountable for all aspects of the work in ensuring that questions related to the accuracy or integrity of any part of the work are appropriately investigated and resolved. All persons designated as authors qualify for authorship, and all those who qualify for authorship are listed.

## CONFLICT OF INTEREST

The authors declare no conflicts of interest.

## FUNDING INFORMATION

No financial assistance was used to conduct the study described in the manuscript or used to assist with the preparation of the manuscript.

## Supporting information

Statistical Summary Document

## Data Availability

The datasets generated during and analysed during the current study are available from the corresponding author upon reasonable request.

## References

[eph13325-bib-0001] Andersen, L. L. , & Aagaard, P. (2006). Influence of maximal muscle strength and intrinsic muscle contractile properties on contractile rate of force development. European Journal of Applied Physiology, 96(1), 46–52. 16249918 10.1007/s00421-005-0070-z

[eph13325-bib-0002] Ansdell, P. , Brownstein, C. G. , Škarabot, J. , Hicks, K. M. , Simoes, D. C. M. , Thomas, K. , Howatson, G. , Hunter, S. K. , & Goodall, S. (2019). Menstrual cycle‐associated modulations in neuromuscular function and fatigability of the knee extensors in eumenorrheic women. Journal of Applied Physiology, 126(6), 1701–1712. 30844334 10.1152/japplphysiol.01041.2018

[eph13325-bib-0003] Attia, M. , & Engel, P. (1981). Thermal alliesthesial response in man is independent of skin location stimulated. Physiology & Behavior, 27(3), 439–444. 7335784 10.1016/0031-9384(81)90329-2

[eph13325-bib-0004] Baker, F. C. , Siboza, F. , & Fuller, A. (2020). Temperature regulation in women: Effects of the menstrual cycle. Temperature, 7(3), 226–262. 10.1080/23328940.2020.1735927 PMC7575238 33123618

[eph13325-bib-0005] Behan, F. P. , Pain, M. T. G. , & Folland, J. P. (2018). Explosive voluntary torque is related to whole‐body response to unexpected perturbations. Journal of Biomechanics, 81, 86–92. 30268357 10.1016/j.jbiomech.2018.09.016

[eph13325-bib-0006] Brown, G. A. , & Williams, G. M. (1982). The effect of head cooling on deep body temperature and thermal comfort in man. Aviation, Space, and Environmental Medicine, 53(6), 583–586. 7115244

[eph13325-bib-0007] Cabanac, M. (1971). Physiological role of pleasure. Science, 173(4002), 1103–1107. 5098954 10.1126/science.173.4002.1103

[eph13325-bib-0008] Cabanac, M. (1975). Temperature regulation. Annual Review of Physiology, 37(1), 415–439. 10.1146/annurev.ph.37.030175.002215 123725

[eph13325-bib-0009] Cohen, J. (1988). Statistical power analysis for the behavioral sciences (2nd ed.) 2.print. ed. Erlbaum.

[eph13325-bib-0010] Cotter, J. D. , & Taylor, N. A. (2005). The distribution of cutaneous sudomotor and alliesthesial thermosensitivity in mildly heat‐stressed humans: An open‐loop approach. The Journal of Physiology, 565(1), 335–345. 15760945 10.1113/jphysiol.2004.081562 PMC1464483

[eph13325-bib-0011] Davies, C. T. , Mecrow, I. K. , & White, M. J. (1982). Contractile properties of the human triceps surae with some observations on the effects of temperature and exercise. European Journal of Applied Physiology and Occupational Physiology, 49(2), 255–269. 6889502 10.1007/BF02334074

[eph13325-bib-0012] de Dear, R. J. , Ring, J. W. , & Fanger, P. O. (1993). Thermal sensations resulting from sudden ambient temperature changes. Indoor Air, 3(3), 181–192.

[eph13325-bib-0013] de Ruiter, C. J. , & de Haan, A. (2000). Temperature effect on the force/velocity relationship of the fresh and fatigued human adductor pollicis muscle. Pflugers Archiv: European Journal of Physiology, 440(1), 163–170. 10864011 10.1007/s004240000284

[eph13325-bib-0014] de Ruiter, C. J. , Jones, D. A. , Sargeant, A. J. , & de Haan, A. (1999). Temperature effect on the rates of isometric force development and relaxation in the fresh and fatigued human adductor pollicis muscle. Experimental Physiology, 84(6), 1137–1150. 10564710 10.1017/s0958067099018953

[eph13325-bib-0015] Dewhurst, S. , Riches, P. E. , Nimmo, M. A. , & De Vito, G. (2005). Temperature dependence of soleus H‐reflex and m wave in young and older women. European Journal of Applied Physiology, 94(5–6), 491–499. 15952024 10.1007/s00421-005-1384-6

[eph13325-bib-0016] Diprose, W. K. , Morgan, C. A. , Wang, M. T. , Diprose, J. P. , Lin, J. C. , Sheriff, S. , Campbell, D. , & Barber, P. A. (2022). Active conductive head cooling of normal and infarcted brain: A magnetic resonance spectroscopy imaging study. Journal of Cerebral Blood Flow and Metabolism, 42(11), 2058–2065. 35707879 10.1177/0271678X221107988 PMC9580175

[eph13325-bib-0017] Domire, Z. J. , Boros, R. L. , & Hashemi, J. (2011). An examination of possible quadriceps force at the time of anterior cruciate ligament injury during landing: A simulation study. Journal of Biomechanics, 44(8), 1630–1632. 21457987 10.1016/j.jbiomech.2011.03.001

[eph13325-bib-0018] Flouris, A. D. , & Cheung, S. S. (2009). Human conscious response to thermal input is adjusted to changes in mean body temperature. British Journal of Sports Medicine, 43(3), 199–203. 18216157 10.1136/bjsm.2007.044552

[eph13325-bib-0019] Folland, J. P. , Buckthorpe, M. W. , & Hannah, R. (2014). Human capacity for explosive force production: Neural and contractile determinants. Scandinavian Journal of Medicine & Science in Sports, 24(6), 894–906. 25754620 10.1111/sms.12131

[eph13325-bib-0020] Gagge, A. P. , Stolwijk, J. A. , & Hardy, J. D. (1967). Comfort and thermal sensations and associated physiological responses at various ambient temperatures. Environmental Research, 1(1), 1–20. 5614624 10.1016/0013-9351(67)90002-3

[eph13325-bib-0021] Gordon, R. J. F. H. , Tillin, N. A. , & Tyler, C. J. (2020). The effect of head and neck per‐cooling on neuromuscular fatigue following exercise in the heat. Applied Physiology, Nutrition, and Metabolism, 45(11), 1238–1246. 10.1139/apnm-2020-0079 32437624

[eph13325-bib-0022] Gordon, R. J. F. H. , Tyler, C. J. , Castelli, F. , Diss, C. E. , & Tillin, N. A. (2021). Progressive hyperthermia elicits distinct responses in maximum and rapid torque production. Journal of Science and Medicine in Sport, 24(8), 811–817. 33775526 10.1016/j.jsams.2021.03.007

[eph13325-bib-0023] Hensel, H. (1981). Thermoreception and temperature regulation. Monographs of the Physiological Society, 38, 1–321. 6820811

[eph13325-bib-0024] Izquierdo, M. , Aguado, X. , Gonzalez, R. , López, J. L. , & Häkkinen, K. (1999). Maximal and explosive force production capacity and balance performance in men of different ages. European Journal of Applied Physiology and Occupational Physiology, 79(3), 260–267. 10048631 10.1007/s004210050504

[eph13325-bib-0025] Krosshaug, T. , Nakamae, A. , Boden, B. P. , Engebretsen, L. , Smith, G. , Slauterbeck, J. R. , Hewett, T. E. , & Bahr, R. (2007). Mechanisms of anterior cruciate ligament injury in basketball: Video analysis of 39 cases. The American Journal of Sports Medicine, 35(3), 359–367. 17092928 10.1177/0363546506293899

[eph13325-bib-0026] Maffiuletti, N. A. , Aagaard, P. , Blazevich, A. J. , Folland, J. , Tillin, N. , & Duchateau, J. (2016). Rate of force development: Physiological and methodological considerations. European Journal of Applied Physiology, 116(6), 1091–1116. 26941023 10.1007/s00421-016-3346-6 PMC4875063

[eph13325-bib-0027] Marino, F. E. (2011). The critical limiting temperature and selective brain cooling: Neuroprotection during exercise? International Journal of Hyperthermia, 27(6), 582–590. 21846194 10.3109/02656736.2011.589096

[eph13325-bib-0028] Martin, P. G. , Marino, F. E. , Rattey, J. , Kay, D. , & Cannon, J. (2005). Reduced voluntary activation of human skeletal muscle during shortening and lengthening contractions in whole body hyperthermia. Experimental Physiology, 90(2), 225–236. 15604113 10.1113/expphysiol.2004.028977

[eph13325-bib-0029] Morrison, S. , Sleivert, G. G. , & Cheung, S. S. (2004). Passive hyperthermia reduces voluntary activation and isometric force production. European Journal of Applied Physiology, 91(5–6), 729–736. 15015001 10.1007/s00421-004-1063-z

[eph13325-bib-0030] Mower, G. D. (1976). Perceived intensity of peripheral thermal stimuli is independent of internal body temperature. Journal of Comparative and Physiological Psychology, 90(12), 1152–1155. 993393 10.1037/h0077284

[eph13325-bib-0031] Nybo, L. , & Nielsen, B. (2001). Hyperthermia and central fatigue during prolonged exercise in humans. Journal of Applied Physiology, 91(3), 1055–1060. 11509498 10.1152/jappl.2001.91.3.1055

[eph13325-bib-0032] Périard, J. D. , Christian, R. J. , Knez, W. L. , & Racinais, S. (2014). Voluntary muscle and motor cortical activation during progressive exercise and passively induced hyperthermia. Experimental Physiology, 99(1), 136–148. 24036591 10.1113/expphysiol.2013.074583

[eph13325-bib-0033] Piil, J. F. , Christiansen, L. , Morris, N. B. , Mikkelsen, C. J. , Ioannou, L. G. , Flouris, A. D. , Lundbye‐Jensen, J. , & Nybo, L. (2020). Direct exposure of the head to solar heat radiation impairs motor‐cognitive performance. Scientific Reports, 10(1), 7812. 32385322 10.1038/s41598-020-64768-w PMC7210303

[eph13325-bib-0034] Raccuglia, M. , Heyde, C. , Lloyd, A. , Ruiz, D. , Hodder, S. , & Havenith, G. (2018). Anchoring biases affect repeated scores of thermal, moisture, tactile and comfort sensations in transient conditions. International Journal of Biometeorology, 62(11), 1945–1954. 30083800 10.1007/s00484-018-1595-2 PMC6182318

[eph13325-bib-0035] Racinais, S. , Gaoua, N. , & Grantham, J. (2008). Hyperthermia impairs short‐term memory and peripheral motor drive transmission. The Journal of Physiology, 586(19), 4751–4762. 18703579 10.1113/jphysiol.2008.157420 PMC2607529

[eph13325-bib-0036] Ramanathan, N. L. (1964). A new weighting system for mean surface temperature of the human body. Journal of Applied Physiology, 19(3), 531–533. 14173555 10.1152/jappl.1964.19.3.531

[eph13325-bib-0037] Rattey, J. , Martin, P. G. , Kay, D. , Cannon, J. , & Marino, F. E. (2006). Contralateral muscle fatigue in human quadriceps muscle: Evidence for a centrally mediated fatigue response and cross‐over effect. Pflugers Archiv: European Journal of Physiology, 452(2), 199–207. 16365782 10.1007/s00424-005-0027-4

[eph13325-bib-0038] Ross, E. Z. , Cotter, J. D. , Wilson, L. , Fan, J. L. , Lucas, S. J. , & Ainslie, P. N. (2012). Cerebrovascular and corticomotor function during progressive passive hyperthermia in humans. Journal of Applied Physiology, 112(5), 748–758. 22134692 10.1152/japplphysiol.00988.2011

[eph13325-bib-0039] Saboisky, J. , Marino, F. E. , Kay, D. , & Cannon, J. (2003). Exercise heat stress does not reduce central activation to non‐exercised human skeletal muscle. Experimental Physiology, 88(6), 783–790. 14603378 10.1113/eph8802611

[eph13325-bib-0040] Schlader, Z. J. , Simmons, S. E. , Stannard, S. R. , & Mündel, T. (2011a). The independent roles of temperature and thermal perception in the control of human thermoregulatory behavior. Physiology & Behavior, 103(2), 217–224. 21315099 10.1016/j.physbeh.2011.02.002

[eph13325-bib-0041] Schlader, Z. J. , Simmons, S. E. , Stannard, S. R. , & Mündel, T. (2011b). Skin temperature as a thermal controller of exercise intensity. European Journal of Applied Physiology, 111(8), 1631–1639. 21197543 10.1007/s00421-010-1791-1

[eph13325-bib-0042] Sunderland, C. , Stevens, R. , Everson, B. , & Tyler, C. J. (2015). Neck‐cooling improves repeated sprint performance in the heat. Frontiers in Physiology, 6, 314. 26594177 10.3389/fphys.2015.00314 PMC4633514

[eph13325-bib-0043] Thomas, M. M. , Cheung, S. S. , Elder, G. C. , & Sleivert, G. G. (2006). Voluntary muscle activation is impaired by core temperature rather than local muscle temperature. Journal of Applied Physiology, 100(4), 1361–1369. 16339343 10.1152/japplphysiol.00945.2005

[eph13325-bib-0044] Tillin, N. A. , Jimenez‐Reyes, P. , Pain, M. T. , & Folland, J. P. (2010). Neuromuscular performance of explosive power athletes versus untrained individuals. Medicine and Science in Sports and Exercise, 42(4), 781–790. 19952835 10.1249/MSS.0b013e3181be9c7e

[eph13325-bib-0045] Tillin, N. A. , Pain, M. T. , & Folland, J. (2013a). Explosive force production during isometric squats correlates with athletic performance in rugby union players. Journal of Sports Sciences, 31(1), 66–76. 22938509 10.1080/02640414.2012.720704

[eph13325-bib-0046] Tillin, N. A. , Pain, M. T. , & Folland, J. P. (2012). Contraction type influences the human ability to use the available torque capacity of skeletal muscle during explosive efforts. Proceedings of the Royal Society B, 279(1736), 2106–2115. 22258636 10.1098/rspb.2011.2109 PMC3321696

[eph13325-bib-0047] Tillin, N. A. , Pain, M. T. , & Folland, J. P. (2013b). Identification of contraction onset during explosive contractions. Response to Thompson et al. “Consistency of rapid muscle force characteristics: Influence of muscle contraction onset detection methodology” [J electromyogr kinesiol 2012;22(6):893‐900. Journal of Electromyography and Kinesiology, 23(4), 991–994. 23742916 10.1016/j.jelekin.2013.04.015

[eph13325-bib-0048] Tillin, N. A. , Pain, M. T. G. , & Folland, J. P. (2018). Contraction speed and type influences rapid utilisation of available muscle force: Neural and contractile mechanisms. The Journal of Experimental Biology, 221(Pt 24). 10.1242/jeb.193367 30348648

[eph13325-bib-0049] Todd, G. , Butler, J. E. , Taylor, J. L. , & Gandevia, S. C. (2005). Hyperthermia: A failure of the motor cortex and the muscle. The Journal of Physiology, 563(2), 621–631. 15613373 10.1113/jphysiol.2004.077115 PMC1665582

[eph13325-bib-0050] Tyler, C. J. , & Sunderland, C. (2011a). Cooling the neck region during exercise in the heat. Journal of Athletic Training, 46(1), 61–68. 21214352 10.4085/1062-6050-46.1.61 PMC3017491

[eph13325-bib-0051] Tyler, C. J. , & Sunderland, C. (2011b). Neck cooling and running performance in the heat: Single versus repeated application. Medicine and Science in Sports and Exercise, 43(12), 2388–2395. 21606877 10.1249/MSS.0b013e318222ef72

[eph13325-bib-0052] Tyler, C. J. , Wild, P. , & Sunderland, C. (2010). Practical neck cooling and time‐trial running performance in a hot environment. European Journal of Applied Physiology, 110(5), 1063–1074. 20694731 10.1007/s00421-010-1567-7

[eph13325-bib-0053] Young, A. J. , Sawka, M. N. , Epstein, Y. , Decristofano, B. , & Pandolf, K. B. (1987). Cooling different body surfaces during upper and lower body exercise. Journal of Applied Physiology, 63(3), 1218–1223. 3654466 10.1152/jappl.1987.63.3.1218

